# Exploiting node metadata to predict interactions in bipartite networks using graph embedding and neural networks

**DOI:** 10.1098/rsos.220079

**Published:** 2022-08-24

**Authors:** Rogini Runghen, Daniel B. Stouffer, Giulio V. Dalla Riva

**Affiliations:** ^1^ Centre for Integrative Ecology, School of Biological Sciences, University of Canterbury, Christchurch, New Zealand; ^2^ School of Mathematics and Statistics, University of Canterbury, Christchurch, New Zealand; ^3^ The Roux Institute, Northeastern University, Boston, MA, USA; ^4^ Khoury College of Computer Sciences, Northeastern University, Boston, MA, USA

**Keywords:** Random Dot Product Graphs, machine learning, link prediction, metadata, predictive models, graph embedding

## Abstract

Networks are increasingly used in various fields to represent systems with the aim of understanding the underlying rules governing observed interactions, and hence predict how the system is likely to behave in the future. Recent developments in network science highlight that accounting for node metadata improves both our understanding of how nodes interact with one another, and the accuracy of link prediction. However, to predict interactions in a network within existing statistical and machine learning frameworks, we need to learn objects that rapidly grow in dimension with the number of nodes. Thus, the task becomes computationally and conceptually challenging for networks. Here, we present a new predictive procedure combining a statistical, low-rank graph embedding method with machine learning techniques which reduces substantially the complexity of the learning task and allows us to efficiently predict interactions from node metadata in bipartite networks. To illustrate its application on real-world data, we apply it to a large dataset of tourist visits across a country. We found that our procedure accurately reconstructs existing interactions and predicts new interactions in the network. Overall, both from a network science and data science perspective, our work offers a flexible and generalizable procedure for link prediction.

## 1. Introduction

Real-world network datasets are often largely incomplete owing to non-exhaustive sampling or the presence of complex hidden processes making data collection difficult [1,2]. As a result, accounting for incompleteness within data (e.g. ‘missing links’) is of key importance both to understand how different components in a system interact with one another and to accurately predict future trends in a system [3–5]. The act of predicting ‘missing links’ or new links in a network is referred as link prediction in various fields [5,6]. The most commonly used methods to tackle link prediction include topological approaches, block model-based methods and graph-embedding methods. In topological methods, certain metrics describing the structure of a network (e.g. network properties such as node degree and various centrality measures) are used to predict interactions [6]. Block model-based approaches, such as the probabilistic generative family of Stochastic Block Models (SBMs) (and variants), aggregate nodes into groups based on their similarity of interactions [7–11]. Graph embedding methods on the other hand rely on projecting nodes onto an abstract latent feature space, so that the interaction probabilities depend on these latent features [12–14].

Multiple studies have shown that incorporating node metadata as covariates can both deepen our understanding of the network structure [15–18], and improve link prediction accuracy in networks [17,19–21]. However, incorporating node metadata presents various challenges. For instance, metadata diversity—i.e. whether the metadata variables are categorical or continuous—may require different modelling frameworks [20,22]. Owing to the high number of nodes in large networks, they can be considered as high-dimensional objects: indeed, when the network is represented as a matrix, each node is an additional coordinate. Therefore, accounting for metadata at the node level may also make the computational requirements overly demanding, as the complexity of the problem scales with the square of the number of nodes. To date, most attempts to incorporate node metadata for link prediction purposes have focused on node-aggregating methods such as SBMs and its variants [16,19,20,23]. These methods make the prediction task more amenable by aggregating nodes into homogeneous groups. However, by doing so, they assume that all nodes within one group behave according to the same interaction probabilities, and thus are statistically indistinguishable [7,8]. Unfortunately by disregarding the heterogeneity of interactions observed at the node level, such approaches oversimplify the network data. Here, we instead focus on using graph embedding methods which allow us to predict the interaction probabilities of each node directly, rather than aggregating the nodes in groups.

In our current study, we propose a new procedure that combines a graph embedding method with machine learning to predict interactions from node metadata. As the functional relationship between node metadata and the abstract latent feature spaces of a network is often unknown prior to data inspection, and can be very complicated, here we suggest using machine learning techniques to find an accurate mapping. In our procedure, we first use the graph embedding method to project nodes of the observed network on an abstract latent feature space at a lower-dimensional space. By doing so, it allows us to learn a mapping from the node metadata to their abstract latent feature space (that we infer from the observed network) in an adequately low-dimensional space. Because we move the problem from the original graph space to a lower dimensional latent feature space, our procedure could potentially simplify the task of predicting interaction in large networks. Here, we specifically used neural networks as our machine learning technique to relate the observed node metadata onto the latent feature spaces of the observed network. The high flexibility of neural networks allowed us to account for the diversity of metadata. To illustrate the application of the proposed procedure in predicting interactions, we used a large dataset of tourist visits to destinations across New Zealand. Overall, our results showed that the proposed procedure accurately predicts interactions in bipartite networks using both the knowledge from the observed network and the node metadata. Moreover, the proposed procedure also allowed us to predict interactions for new nodes.

## 2. Material and methods

In this article, we focus on bipartite networks—i.e. networks that feature nodes of two types and with interactions (or links) that only occur between the different set of nodes. In the following sections, we describe: (i) the adopted network modelling procedure: first describing the Random Dot Product Graphs (RDPG) model, then explaining how to infer, from an observed network, the position of its nodes in the latent feature space, and finally how to relate the node metadata to the nodes in their latent feature space using a machine technique; (ii) an application to empirical network data; and (iii) the sensitivity and performance analyses we conducted to validate the proposed procedure.

### 2.1. Extending the Random Dot Product Graphs model to bipartite networks

RDPGs are a class of latent position models [24] developed originally to analyse social networks [25,26], and then extended to many other applications and types of networks [13,22,27–29]. To describe interactions in a network, such models assume that the probability of observing an interaction between two nodes is a function of the nodes’ features [25,26]. Here, we specifically use the RDPG implementation of Young & Scheinerman [25] to predict interactions in a bipartite context.

We define a bipartite network *G* as two distinct sets of nodes, *V* and *P* containing *M* and *N* nodes, respectively, that is where *V* = {*v*_1_, …, *v*_*n*_} and *P* = {*p*_1_, …, *p*_*m*_}, respectively; and a set of links, *E*, between the sets of nodes—i.e. (*v*_*i*_, *p*_*j*_) ∈ *E*. We denote such a bipartite network as *G*(*V*, *P*, *E*). The bipartite network can be further represented as an adjacency matrix, where *G* is represented as the matrix *A* ∈ {0, 1}^*M*×*N*^, where *A*_*ij*_ = 1 if (*v*_*i*_, *p*_*j*_) ∈ *E* and *A*_*ij*_ = 0 otherwise.

In a bipartite RDPG model, each node *v*_*i*_ and *p*_*j*_ is assigned a vector of latent features $x_{i}\in R^{d}$ and $y_{\;j}\in R^{d}$. We call *d* the dimension of the network’s latent feature space, and the vectors *x*_*i*_ and *y*_*j*_ indicate the positions of the nodes *v*_*i*_ and *p*_*j*_, respectively, in the network’s latent feature space. The bipartite RDPG model further treats links as independent Bernoulli variables: two nodes interact with a probability equal to the dot product of their latent vectors, in the formula

2.1 $$Pr((v_{i},p_{\;j})\in E)=x_{i}\cdot y_{\;j}.$$

In matrix notation, we can represent the latent positions of all the nodes in *V* and *P* as the rows of a matrix **V** and the columns of a matrix **P**, respectively. As a result, the matrix of probabilities of interactions between the two node types in the network can be written as the matrix product **VP**.

For the matrix product to have meaning, the two matrices **V** and **P** need to have compatible dimension, which is satisfied if the latent feature spaces for *V* and *P* are equidimensional (that is, if the vectors *x*_*i*_ and *y*_*j*_ have the same number of coordinates). Moreover, for the matrix product to represent probabilities, the products must be in the [0, 1] range, which imposes additional geometric constraints on the latent feature spaces [14]. Lastly, it is worth noting that any orthogonal transformation—e.g. a rotation—applied to both **V** and **P** would result in an equivalent matrix of interaction probabilities: this will limit us to be able to infer the latent feature spaces up to an orthogonal transformation. Thus, we should refrain from reading any meaning in the absolute position of a node in the latent feature space.

### 2.2. Inferring the position of nodes in the latent feature space

In theory, neither the nodes’ positions in the latent feature space, nor the dimension of the latent feature space are observable. Thus, we need to infer them from the observed network. To do so, we can exploit the adjacency spectral embedding—which is the truncated singular value decomposition (SVD) of a network adjacency matrix—to obtain an unbiased estimate of the nodes’ positions in the network’s latent feature spaces [29].

The full rank SVD of the observed adjacency matrix *A* is given by three matrices *L*, Σ and *R* such that $A=L\times \Sigma \times R^{T}$, with *L* and *R* real orthogonal matrices, and Σ a diagonal matrix whose entries are the singular values of *A* in decreasing order. As the sets of nodes *V* and *P* contain *M* and *N* nodes, respectively, the matrices *L*, Σ and *R* will have dimensions *M* × *S*, *S* × *S* and *N* × *S*, respectively, where *S* = min (*M*, *N*). To compute the SVD of a matrix, we used the default svd function in R [30], which performed well for the large visitation dataset described in later sections (Other fast algorithms that allow the decomposition of very large matrices such as in Liang *et al.* [31] and Zhou & Li [32] also exist if needed).

Let *d* be the chosen dimension for the observed network latent feature space. We denote $\hat{L}$, $\hat{\Sigma }$ and $\hat{R}$ as the *d*-truncations of *L*, Σ and *R*, respectively. We obtain them by retaining all the rows and the first *d* columns of *L* and *R*, and the first *d* rows and columns of Σ. We then compute the *d*-dimensional bipartite adjacency embedding of *A* as

2.2 $$\begin{array}{c} \begin{array}{r} V\approx \hat{V}=\hat{L}\sqrt{\hat{\Sigma }}\\ P\approx \hat{P}=\sqrt{\hat{\Sigma }}{\hat{R}}^{T}, \end{array}\}\;\text{and} \end{array}$$

where $\sqrt{\hat{\Sigma }}$ is a *d* × *d* diagonal matrix defined by the square root of the *d* greatest singular values of *A*. Note that as we are interested in truncated SVD calculated at an adequate *d* as in Athreya *et al.* [14], all the results from the dot products are kept within the range of [0, 1]—i.e. all values below zeroes are treated as zeroes, and all values above ones are treated as ones. As such, one can interpret them as probabilities. We specifically used the profile-likelihood criterion of Zhu & Ghodsi [33] to estimate an adequate dimension *d* ≤ *S* for the latent feature space.

### 2.3. Relating node metadata to the latent feature space to predict interactions in a network

Given the positions of all nodes in the latent feature spaces, the RDPG model completely determines the interaction probabilities between all nodes in the network. This implies that, if we were able to go from a node’s metadata to its position in the network’s latent feature space, we would be able to estimate its interaction probabilities with all other nodes in the network.

Let us consider the simplest scenario where we have a one-dimensional latent feature space (in other words, the latent feature space is a vector with one coordinate, *d* = 1) and real-valued metadata for the nodes in *V* and *P*. We can learn a mapping from the metadata to the latent feature space by fitting a linear regression model with the positions of nodes *v*_*i*_ in the latent feature space, $x_{i}\in R$, as dependent variables and the metadata vectors *m*_*i*_ as independent predictor variables, provided that we have at least as many nodes as predictors. Let *β*_0_ and *β* be the estimated intercept and vector of slope parameters for the linear model. The resulting model allows us to get the position of node *v*_*i*_ in the predicted latent feature space: $x_{i}^{*}\in R$. We can then estimate the interaction probabilities of *v*_*i*_ via the dot product of $x_{i}^{*}$ with the positions of the nodes in *P*’s latent feature space. Similarly, we can use the inferred linear model to predict the latent features of a *new* node *v*_*n*+1_ added to the network—i.e. a node which was previously not observed—from the node’s metadata as follows: $x_{n+1}^{*}=\beta_{0}+m_{n+1}\cdot \beta$. Then, to estimate the interaction probabilities of *v*_*n*+1_, we proceed with the dot product of *x*_*n*+1_ with the positions of the nodes in *P*’s latent feature space.

The latent feature space of large empirical networks is multivariate (even if not necessarily large, 1 < *d* ≪ min (*M*, *N*)). In general, node metadata are of different types—i.e. categorical and continuous. Finally, the relationship between node metadata and latent feature spaces are often nonlinear. Fortunately, a variety of statistical and machine learning approaches exist to solve the task of predicting a *d*-dimensional real-valued vector from another vector (potentially larger and mixed valued). In particular, neural networks approaches can be used [34,35]. In our application, we compare the performance of a classic linear regression, using ordinary least squares, and different neural network architectures.

**Figure 1.**
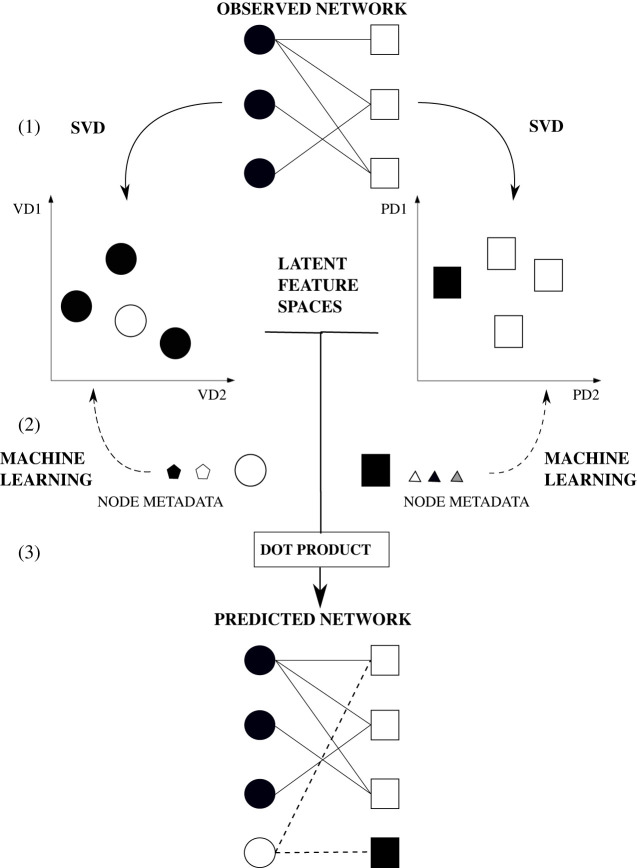
Using a Random Dot Product Graphs framework to predict interactions in a bipartite network. Note that here we use our case study—i.e. the travelling patterns of visitors to touristic destinations—to illustrate the framework. We first consider the travelling patterns of visitors as network data. In (1), the bipartite representation of travelling patterns of visitors: nodes are of two types—circles represent visitors and squares represent places, and links indicate a trip travelled by a given visitor to a given place. Here, the solid lines represent the observed links. In (2), we first estimate the position of nodes within the observed bipartite network using a singular value decomposition (SVD) on the adjacency matrix representing the visitor–place interaction matrix. As a result, we obtain two latent feature spaces: a visitor latent feature space and place latent feature space. Note that here we show the embeddings of nodes of both visitors and places, respectively, in a latent feature space of dimension *d* = 2: LD1 and LD2. In (3), we then relate the node metadata directly to their latent feature spaces. To do so, we use machine learning techniques to find the relationship between the two. Step (3) allows us to reconstruct interactions between observed nodes (visitors and places, respectively). To further predict *new* interactions in the network using observed metadata of new visitors and new places, respectively—the models used to find the relationship of the node metadata to the latent feature spaces are used. By doing so, we can project the new visitor and new place into the respective latent feature spaces LD1 and LD2. Finally, in (4), using the dot product, we are able to predict the probability of interaction between the new visitor and the new place added to the visitation network. Here, the dashed lines represent the new predicted links for observed and new nodes.

To conclude, we have shown that one can use (i) a truncated SVD to estimate the latent feature spaces of nodes in a bipartite network, (ii) a variety of statistical and machine learning approaches to predict the latent features from the node metadata, and (iii) a simple dot product to predict interaction probabilities from nodes’ latent features (figure 1). In the next section, we apply our procedure to a large bipartite network of tourist–place visitation data deploying both linear regression models and two different neural network architectures.

### 2.4. Application to real-world data: predicting tourist destinations from visitors and places metadata

To test the presented procedure, in this section, we describe its application to visitation data representing the travel destinations of tourists across New Zealand. We specifically show how one can use visitor and place metadata, respectively, to estimate the interaction probabilities for visitors to travel to places within the country (or the likelihood for places to be visited). By doing so, this allowed us to both predict the likelihood for new visitors to travel to existing destinations (observed nodes in the network), and the likelihood for visitors (existing or new) to travel to new places (or new tourist attractions/travel destinations). As such, we show how the proposed procedure allows us to reconstruct the observed visitation network. Moreover, we show how one can potentially use the proposed procedure to predict *new* interactions—i.e. when new visitors and places are added to the visitation network—using only the new node metadata and knowledge from the existing network.

### 2.5. Visitation data

To get an overview of the visitor travelling patterns across New Zealand, we extracted data from two national surveys conducted by the New Zealand Ministry of Business, Innovation and Employment (MBIE): The International Visitor Survey [36] and the Domestic Travel Survey [37]. The International Visitor Survey targeted international visitors departing New Zealand at the four main international airports (Auckland, Wellington, Christchurch and Queenstown), whereas the Domestic Travel Survey contacted domestic travellers via phone interviews about their recent trips. Both surveys record the list of places to which each visitor travelled during their trip within New Zealand. This accounted for a total of 189 942 visitors travelling to 2616 places across the country. Note that these numbers refer to *only* visitor and places for which the complete set of metadata were available (electronic supplementary material, table S1).

### 2.6. Node attributes: visitor and place metadata

Visitor metadata includes age, gender, activity type and the mode of transportation used during their trip (table 1).

**Table 1.** Summary of node metadata used.

node type	metadata	data type	classes
visitor	gender	categorical	male, female
visitor	age	categorical	age group: <20, 21–25, 26–34, 35–39, 40–44, 45–49, 50–51, 64–69, >70
visitor	activity type	categorical	hiking, site-seeing, water activities, museums and other heritage sites, visiting family, work purposes
visitor	mode of transportation	categorical	car, van, boat, tour bus, bus, helicopter, aeroplane
place	place geolocation	continuous	latitude and longitude of locations
place	place type	categorical	heritage site, crown protected area, town, village, recreational site
place	regional council	categorical	Northland, Auckland, Waikato, Bay of Plenty, Gisborne, Hawke’s Bay, Taranaki, Manawatu-Wanganui, Wellington, Tasman/Nelson, Marlborough, West Coast, Canterbury, Otago, Southland, and areas outside regional council

These characteristics are present for both the Domestic Travel Survey and the International Visitor Survey. As the survey data only had the name of places visited by travellers, we had to define the attributes of the different places within the visitation data. In general, places across New Zealand can be categorized based on the ownership of the land or the type of activities performed on those lands. Therefore, to identify the land type of each place within the visitation data, we used the geospatial maps provided by Land Information New Zealand [38] to map the places extracted from the different national surveys. For example by doing the latter, this allowed us to distinguish whether a particular place was categorized as a recreational site or national heritage site (table 1).

### 2.7. Predicting visitor–place interactions in visitation network using node metadata

Here, we are particularly interested in testing our predictive procedure in two different contexts. First, predicting the probabilities of missing interactions of in-sample nodes present in the observed network and for which we have node metadata, which we henceforth refer to as ‘observed’ nodes. Second, predicting the probabilities of missing interactions of out-of-sample nodes for which we only have the node metadata, which we henceforth refer as ‘new’ nodes. As such, we split the visitation data based on the visitor–place interactions, as well as in-sample and out-of-sample nodes, into a training and a validation set. The training set contains only *observed* nodes and in-sample visitor–place interactions; the validation set contains a mixture of *observed* and *new* nodes, both of which will feature out-of-sample visitor–place interactions as stated in table 2.

**Table 2.** Summary of number of visitors and places used for the different steps of the predictive procedure.

dataset	analysis	no. visitors	no. places	visitor–place interactions
training set (70% of full dataset)	SVD	101 656	430	421 784 625
model training set (50% of training set)	neural network	71 159	301	210 892 312
model test set (50% of training set)	neural network	30 497	129	210 892 313
validation set (30% of full dataset)	neural network, dot product	88 286 (43 662 *new*)	360 (120 *new*)	140 594 875

Note that to make sure that part of the validation set served as new data—i.e. for *new* visitors and places, respectively—we made sure that the validation set contained both visitors and place identities not present in the training set. Artificially removing nodes from the observed network allows us to further test the ability of the proposed predictive procedure to predict interaction probabilities of *new* nodes—i.e. when either new visitors or places are added to the network—using only their metadata and information from the observed visitation network which can be thought of as the cold-start problem in recommender systems. By including both the *observed* and *new* nodes in the validation set, it allows us to validate our predictive framework. More specifically, it allows us to verify whether our predictive framework is able to predict out-of-sample interactions for observed nodes within the observed network and to predict out-of-sample interactions of new nodes if they were to be added in the network using their node metadata.

In the rest of this section, we explain our predictive procedure in detail. The procedure involves three key steps: (1) we use the training set to perform an SVD of the adjacency matrix of the observed network to compute the positions of the observed nodes in their latent feature space; (2) we use the model training set to fit regression models that predict the nodes’ positions in the latent feature space as a function of the node metadata, and we validate this process with the model test set; and (3) then, we use the fitted regression models to predict the positions of the nodes from the validation set in their latent feature space; and we use these predicted positions to estimate the interaction probabilities of nodes in the validation set.

(1) We computed the SVD of the training network’s adjacency matrix *A*_*T*_.

Then, truncating the SVD, we computed the positions of the observed visitor and place nodes in their respective latent feature spaces, ${\hat{V}}_{T}$ and ${\hat{P}}_{T}$, respectively:

2.3 $$A_{T}\approx \hat{L}\sqrt{\hat{\Sigma }}\times \sqrt{\hat{\Sigma }}{\hat{R}}^{T}\;:={\hat{V}}_{T}{\hat{P}}_{T}.$$

(2) To predict the nodes’ positions from the node metadata, we fit three different types of multivariate regression models on the training set. Let **v**_*T*_ and **p**_*T*_ be the metadata for visitor and places nodes in the training set, then the regression modelling task is to find a pair of functions $\bar{\;f}$ and $\bar{g}$ such that $\bar{\;f}(v_{T})$ and $\bar{g}(p_{T})$ best approximate ${\hat{V}}_{T}$ and ${\hat{P}}_{T}$, respectively, where $\bar{\;f}$ and $\bar{g}$ are part of some family of functions $\bar{\;f}\in \{f\}$ and $\bar{g}\in \{g\}$. For the sake of clarity, $\bar{\;f}$ and $\bar{g}$ are functions from the space of metadata (visitor node and place node metadata, respectively) to the space of latent features (for visitors and places, respectively).

We fit: (i) a linear regression (baseline)—where we used a linear function to relate directly the metadata to the latent feature space (specified as in the electronic supplementary material, table S3); (ii) a multilayer perceptron (MLP)—i.e. a neural network with one dense hidden layer of 200 nodes using a rectified linear unit (ReLU) as our activation function; and (iii) a neural network with two dense hidden layers (NN) of 250 nodes each and the ReLU activation function. Owing to the variety of data types—i.e. varying from categorical to continuous variables (electronic supplementary material, table S2)—and the high flexibility of neural networks in solving regression and classification problems [39], we compared two learning rates: a constant learning rate of 0.01 and a time-based decay—where the initial learning rate (0.01) decreased by 0.0001 after each epoch. We used the mean absolute error to measure the distance between the predicted and estimated latent features and to assess the accuracy of the model training (refer to the electronic supplementary material, table S4 to see the results obtained when the accuracy of the models were computed using other metrics). To monitor the training of the different models and ensure that they were not overfit, we split the training set into two sets (table 2): a model training set (50% of the training set) and a model test set (50% of the training set)—which serves as a means to validation of the different regression models.

We trained all the regression models on the model training set and evaluated their accuracy on the model test set. We used Google’s deep learning software TensorFlow [40] and Keras [41] implemented in Python 2.7 [42] to fit all the aforementioned models using the adaptive moment estimation (Adam) optimizer [43] with 30 epochs and a batch size of 20. We then used the fitted multivariate regression models to predict the positions of the nodes from the validation dataset in the latent feature space, ${\bar{V}}_{V}$ and ${\bar{P}}_{V}$, respectively. The predicted values ${\bar{V}}_{V}$ and ${\bar{P}}_{V}$ are functions of the node metadata:

2.4 $$\begin{array}{c} \begin{array}{r} {\bar{V}}_{V}\;:=\bar{\;f}(v_{V})\\ {\bar{P}}_{V}\;:=\bar{g}(p_{V}), \end{array}\}\;\text{and} \end{array}$$

where **v**_*V*_ and **p**_*V*_ are the metadata for visitor and places nodes in the validation set, and $\bar{\;f}$ and $\bar{g}$ are the function obtained from the training set.

(3) Using the nodes’ positions ${\bar{V}}_{V}$ and ${\bar{P}}_{V}$ predicted by the models in step (2), we estimated the interaction probabilities for all nodes present in the validation set and the nodes in the training set by multiplying the matrices containing the nodes’ position in their respective latent feature spaces:

2.5 $$\begin{array}{c} \begin{array}{r} \mathrm{Pr}((v_{V},p_{T})\in E)\;:={\bar{V}}_{V}{\hat{P}}_{T}\\ \mathrm{Pr}((v_{T},p_{V})\in E)\;:={\bar{V}}_{T}{\hat{P}}_{V} \end{array}\}\;\text{and} \end{array}$$

where, with some abuse of notation, Pr((*v*_*V*_, *p*_*T*_) ∈ *E*) and Pr((*v*_*T*_, *p*_*V*_) ∈ *E*) are the matrices of interaction probabilities between visitor nodes in the validation set and places nodes in the training set, and between visitor nodes in the training set and places nodes from the validation set. Note that similarly, we can estimate the interaction probabilities for all nodes present within the training set—i.e. Pr((*v*_*T*_, *p*_*T*_) ∈ *E*).

Specific pairwise interaction probabilities can be estimated by multiplying the vector of the predicted latent feature position of new nodes (inferred from the regression methods) to the latent features position vectors (estimated from the SVD) for all observed nodes present in the observed network, that is, using the dot product. For example, considering a new visitor node *n* + 1 with metadata **v**_*n*+1_, a predictive function $\bar{\;f}$, and a known place node *j* whose position in the latent feature space (as obtained by SVD in step 1) is *y*_*j*_, the interaction probability between *n* + 1 and *j* is

2.6 $$\mathrm{Pr}((v_{n+1},j)\in E)\;:=\bar{\;f}(v_{n+1})\cdot y_{j}.$$

### 2.8. Sensitivity and performance analysis of predictive procedure

To validate the proposed predictive framework, we were particularly interested in assessing the performance of the following steps: (i) relating node metadata to the corresponding latent feature spaces (which we refer as the node metadata validation), and (ii) predicting out-of-sample interactions for both observed nodes and new nodes (which we refer as the link prediction validation). As such, we carried out two validations. The node metadata validation was carried out entirely on the training set. The main purpose of the node metadata validation was to ensure that the regression models were accurately relating the node metadata to the corresponding latent feature spaces. To do so, the training set was split into the model training set and model test set (table 2). The model training set was used to train the regression models whereas the model test was used to validate the regression models.

To assess the overall performance of our proposed predictive procedure, we carried out a link prediction validation using the validation dataset. To do so, we calculated the probability of interaction between the nodes in the validation set as

2.7 $$\mathrm{Pr}((v_{\mathrm{validation}},p_{\mathrm{validation}})\in E)\;:={\bar{V}}_{\mathrm{validation}}{\bar{P}}_{\mathrm{validation}}=\bar{\;f}(v_{\mathrm{validation}})\bar{g}(p_{\mathrm{validation}}),$$

where *v*_validation_ and *p*_validation_ are the nodes in the validation set, ${\bar{V}}_{\mathrm{validation}}$ and ${\bar{P}}_{\mathrm{validation}}$ are the predicted latent features positions, **v**_validation_ and **p**_validation_ are the nodes metadata, and $\bar{\;f}$ and $\bar{g}$ are the trained predictive functions. To assess the performance of the overall predictive procedure, we calculated the sensitivity—i.e. the ratio of correctly predicted links to observed links, and the accuracy—i.e. the ratio of correctly predicted observed links (true positive) and correctly absent links (true negative)—in our validation set [44].

Furthermore, to evaluate and assess the performance of the different combinations of RDPG-regression models in correctly predicting the observed interactions, we used the area under curve-receiver operator curve (AUC-ROC) to evaluate the performance of the different combinations. To do so, we calculated the rate of true positives—i.e. predicting an interaction when it is actually present—and false positives—i.e. predicting an interaction when it is actually absent—at different thresholds varying from 0 to 1. AUC-ROC is used as a measure to assess the ability of different models to distinguish between a true positive and a false positive. For instance when AUC=1, the predictive model is able to perfectly distinguish between all the true positive and the true negatives. However, if AUC = 0, then the predictive model is performing as worse as possible—i.e. it is predicting all true negatives as positives (termed as false positives), and all true positives as negatives (termed as false negatives). When 0.5 < AUC ≤ 1, there is a high chance that the predictive model is performing well in distinguishing the true positives from the false positives. When AUC = 0.5, then the predictive model is not able to distinguish between true positives and true negatives—i.e. the predictive model is either predicting randomly true positives as positives and true negatives as negatives or constantly predicting true positives as negatives and true negatives as positives. Thus, the lower the AUC value, the lower the ability for the predictive model to distinguish true positives and true negatives. To quantify the uncertainty associated with the various predictive models we considered, we randomly selected a sample of 1000 observations from the validation set. This subset, which we called the bootstrap dataset is then used to evaluate the various predictive models. We computed the AUC at 95% confidence interval (CI) with 2000 stratified bootstrap replicates. These results provide an indication of the variance of the models performance. Note that the sampling is performed with replacement.

## 3. Results

**Figure 2.**
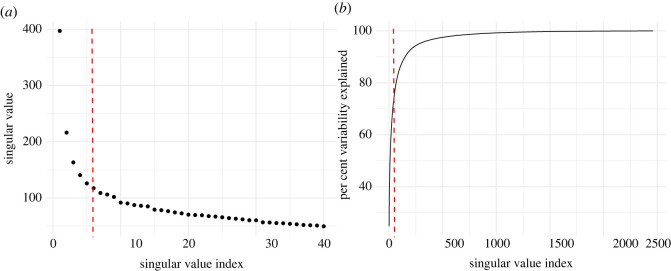
Identifying an adequate dimension *d* of network data. (*a*) The scree plot represents the singular values of the adjacency matrix of the visitation network in decreasing order of *d*. The *x*-axis shows the singular value index, and the *y*-axis indicates the singular values. (*b*) Cumulative plot showing the percentage variability explained with the increasing singular value indexes. The *x*-axis again shows the singular value index and the *y*-axis indicates the per cent of variance explained. Using Zhu & Ghodsi [33]’s profile-likelihood criterion, we picked *d* = 6 as indicated by the red dotted line. This dimension explains 70% of the variability of the visitation network data.

For the training visitation dataset (number of visitors = 136 910, number of links = 636 497), the Zhu & Ghodsi [33]’s profile-likelihood criterion indicated a six dimensional latent feature space (*d* = 6) as adequate. This accounted for approximately 70% variability of the visitation network data (figure 2).

**Figure 3.**
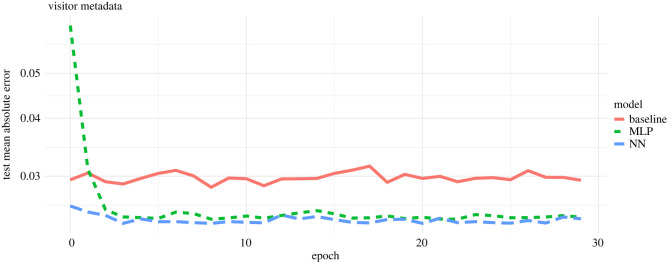
Training of regression models over time when projecting observed visitor metadata onto the latent feature space using an adaptive moment estimation (Adam) optimizer run with 30 epochs and a batch size of 20. The plot shows the model validation—i.e. the subset of training visitation dataset used—to validate the three different models in finding the best mapping from the node metadata to the latent feature space. The *x*-axis indicates the epochs. The *y*-axis indicates the mean absolute error (MAE), which is the cost function used to measure the accuracy of model predictions—i.e. it measures the distance between the estimated latent feature space (SVD) and the predicted latent feature space. The red line shows the learning rate of the linear regression model (baseline), the green line indicates the learning rate of the multilayer perceptron model (MLP), and the blue line indicates the neural network with two hidden layers (NN). The plot shows that both the MLP model and NN model performed better than the baseline model.

Overall we found that the neural networks performed better than the baseline model in finding the best mapping from the node metadata to the latent feature spaces. More specifically, for the visitor metadata, we found that the NN (mean squared error (MSE) = 0.0009) and MLP (MSE = 0.0010) performed better compared to the baseline model (MSE = 0.0014) using a constant learning rate (figure 3). We found similar patterns for the time-based learning rate (electronic supplementary material, figure S1).

**Figure 4.**
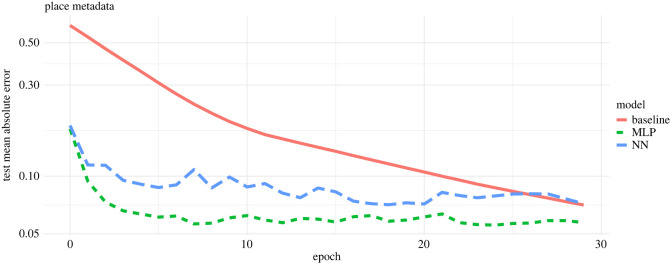
Training of regression models over time when projecting observed place metadata onto the latent feature space using an adaptive moment estimation (Adam) optimizer run with 30 epochs and a batch size of 20. The plot shows the model validation—i.e. the subset of training visitation dataset used—to validate the three different models in finding the best mapping from the node metadata to the latent feature space. The *x*-axis indicates the epochs. The *y*-axis indicates the mean absolute error (MAE), which is the cost function used to measure the accuracy of model predictions—i.e. it measures the distance between the estimated latent feature space (SVD) and the predicted latent feature space. The red line shows the learning rate of the linear regression model (baseline), the green line indicates the learning rate of the multilayer perceptron model (MLP), and the blue line indicates the neural network with two hidden layers (NN). Here the MLP model seems to perform better than the baseline and NN models.

For the place metadata, we found that the MLP model (MSE = 0.125) and linear regression model (MSE = 0.141) performed better than the NN (MSE = 0.143) (figure 4). We also observed similar patterns for models run with the time-based learning rate (electronic supplementary material).

The predictive procedure we proposed performed significantly better than at random—i.e. when compared against AUC = 0.5 (electronic supplementary material, table S5). Comparing the different latent feature prediction models, we found that the dot product of the visitor baseline model and the place MLP performed better with AUC = 0.736, followed by dot product of the visitor MLP and the place MLP model with AUC = 0.701 (table 3).

**Table 3.** Accuracy of model predictions obtained from RDPG-regression procedure. (The table indicates the area under curve (AUC) values for each model calculated using mean absolute error as the cost function to measure the distance between the estimated latent feature spaces and the predicted latent feature space. Note that the value in italics indicates the model with the highest AUC value across all models. The values in brackets are AUC values computed at 95% confidence interval (CI).)

		place		
		baseline	MLP	NN
visitor	baseline	0.630 (95% CI [0.623, 0.625])	*0.736* (95% CI [0.735, 0.795])	0.699 (95% CI [0.596, 0.6002])
	MLP	0.645 (95% CI [0.629, 0.634])	0.701 (95% CI [0.702, 0.715])	0.665 (95% CI [0.617, 0.621])
	NN	0.653 (95% CI [0.630, 0.635])	0.699 (95% CI [0.619, 0.622])	0.670 (95% CI [0.605, 0.609])

## 4. Discussion

In the current study, we present a new predictive procedure which allows us to use both the node metadata and the knowledge gained from the observed network to predict interactions in bipartite networks. Overall, we showed that our proposed predictive procedure works in a real-world context with an accuracy of AUC = 0.736. This indicates that the procedure performed relatively well in distinguishing between true positives and true negatives when predicting interactions. Moreover, we showed that our procedure also allowed us to predict out-of-sample interactions for observed nodes, and more importantly, predict interaction for new nodes added to the network.

To our knowledge, few studies have focused on exploiting node metadata to predict interactions using graph-embedding methods. Most research including node metadata to predict interactions have used node-aggregating methods [16,20,23]. The aforementioned variants of the SBM assume that all nodes belonging to a given group behave identically, ignoring that certain nodes within the given group might be interacting with other nodes in the network to different extents. Note however that recent advances in the field have shown that some SBM variants such as the Mixed Membership Stochastic Block Model [9], or even the degree-corrected SBM [45] can be considered as embedding approaches [29], and can additionally account for the heterogeneity of interactions at the node level [45,46]. However, within the scope of this paper, we did not consider the aforementioned SBM variants. We instead propose using a predictive procedure based on the RDPG model combined with a machine learning algorithm to account for the heterogeneity of interactions observed at the node level. Moreover, using the truncated SVD allows us to represent an observed network at a lower dimension. Then, using the statistical properties of the RDPG model, we can proceed to predict interactions at the node level in a network by simply calculating the dot product of the given node to the other nodes present in the network [27,29,47].

Machine learning techniques such as neural networks are increasingly popular tools in various applications owing to their high predictive accuracy [48,49]. Here, we used neural networks to relate the node metadata to their latent feature spaces obtained from a truncated SVD. Various studies suggest that deeper neural networks—i.e. neural networks with a high number of hidden layers—tend to outperform shallow neural networks in a wide variety of tasks [35,39]. While both of the neural network architectures we tested outperformed the linear regression model in mapping the node metadata onto the latent feature spaces, our results showed that the linear regression model (for the visitors’ metadata) and the neural network with one hidden layer (for the places’ metadata) outperformed the neural network with two hidden layers in predicting links. This therefore suggests that the more complex models are overfitting. Note, however, that the main purpose of our study was *not* to find the absolute best neural network architecture, and hence we do not expect further studies to necessarily confirm this result.

Metadata are known to be good proxies from which to predict interactions in a network [15,17–19,23]. Incorporating node metadata as covariates to network models for link prediction can be tedious. Especially as node metadata can be of varied type—i.e. categorical and continuous variables, and these variables might not have linear relationships to the latent feature space; these factors together necessitate different modelling frameworks [20,22]. Here, we showed that the high flexibility of neural networks (or other machine learning algorithms) enabled the identification of an accurate mapping from the visitors’ metadata onto the visitors’ latent feature space and from the places’ metadata to the places’ latent feature space, respectively. As the functional relationship between node metadata and their position in the latent feature space can be very complicated, neural network methods are a promising approach to learn it. Note that over the past years, a wide suite of methods have been developed to incorporate node metadata as covariates [16,19,23]. It would eventually be important to compare our proposed framework to the existing ones as a benchmark to objectively compare across these different techniques. However, this was beyond the scope of this paper.

While each step of the presented procedure is robust, there might be many sources of error. In the current study, we only present an exploratory analysis of a bipartite network to predict interactions in a visitation network using both node types’ metadata. Rather than attempting to find the optimal dimension of our network data, we instead chose *d* according to the Zhu & Ghodsi's [33] profile-likelihood criterion. We selected *d* *a priori*, based only on the topological structure of the network. It could be interesting to further explore whether using a different procedure to select the dimension of the latent feature space improves the accuracy of link prediction. Indeed, *a posteriori* selection (trying to identify which dimension *d* grants a higher prediction accuracy) is another possibility, but may require substantially greater computational effort.

The way in which we split our training and test set implies that the observed and new node metadata are sampled from the same distribution. Therefore, the models learn a relevant mapping of the new nodes into a suitable region of the latent feature space. However, if this is not the case, and the metadata of the new nodes is completely different from one of the observed nodes, nothing guarantees a good placement in the latent feature space. For instance, in the case of our real-world example, we assumed that the type of visitors (characterized by their node metadata) and their travelling patterns are consistent over time. However, in reality, new visitors with completely different characteristics, with different interests could arrive in the country, which may make using the current proposed framework difficult in predicting their future travelling patterns. How to deal with new nodes with ‘surprising’ metadata is an open problem. Similarly, even if visitors with consistent characteristics were to come, predicting the dynamics of their travelling patterns can be hard as it is highly dependent of the visitors’ behaviour. Accounting for human behaviour has shown to be crucial, especially when predicting their travelling patterns [11,50,51]. For example, seasonal changes, accessibility to places, or even the increasing popularity of particular places owing to social media can influence visitors’ behaviour in travelling across a country [50–52]. Thus, another important aspect that one should consider would be the dynamic nature of the observed network which would influence the link prediction task.

In addition, we assumed all the node metadata could be informative when predicting interactions. As a result, we learnt a mapping from the nodes’ full metadata to their respective latent feature spaces obtained from the truncated SVD. However, we know that not all of the metadata is necessarily informative, especially when predicting interactions [18,53]. Therefore, further investigating the relationship between the node metadata and the latent feature space obtained from the truncated SVD should be done to understand whether certain node metadata are affecting the link prediction accuracy in the presented procedure.

Moving forward, it would be interesting to extend the current procedure to account for missing data in: (i) the interaction probability matrix—i.e. distinguishing new and absent interactions—and (ii) the metadata—i.e. when some of the node metadata are missing. One can imagine a scenario where a survey was carried out, and a person did not complete the full survey. If we were to have the metadata of that particular person, we could potentially interpolate some of their answers. Similarly, in the case where data is extracted from an experimental set-up, data might be missing as a result of failed experiments. Accounting for such missing information can be particularly important. In this direction, deeper or dedicated neural network architectures, such as the ones in Smieja *et al.* [54] and Przewikélikowski *et al.* [55], could be used. More recently, Lerique *et al.* [56] have used a neural network approach to find the joint embedding of metadata and the network structure to predict the interaction probabilities. However, one of the main limitations of the latter approach is the need to find an optimal dimension for the both the node metadata and the network data. Using a machine learning approach to learn a mapping of node metadata directly to their interaction probabilities in large networks remains a hard problem when performed in a very high-dimensional space. Here, we showed that we can simplify that problem by exploiting the properties of a well understood statistical model for complex networks, the RDPG model, and combining it with standard machine learning techniques. The RDPG model grants us a robust estimation of a low-dimensional network embedding (the nodes’ latent feature spaces) and a convenient way to estimate its dimension. As in other examples [57], promising results are obtained not by abandoning a model-based approach to science but by merging it with machine learning techniques.

## Data Availability

All visitation data from the Ministry of Business Innovation & Employment (MBIE) used in this study are publicly available on https://www.mbie.govt.nz/immigration-and-tourism/tourism-research-and-data/tourism-data-releases/ as the International Visitor Survey and the Domestic Travel Survey 1999–2012. All codes used in the current study are available at https://zenodo.org/record/5979635#.Yf7t_y-B3mE. The data are provided in the electronic supplementary material [58].
